# Surgical Hints

**Published:** 1897-02

**Authors:** 


### SURGICAL HINTS.

In septic conditions the patient is often very uncomfortable by reason of dryness of the tongue. A bit of ordinary chewing gum will usually start the oral secretions and will, in a very short time, give relief.

The operation of tracheotomy is rendered much easier by allowing the patient's shoulders to rest upon a thin pillow with no pillow for the head. The tissues at the front of the neck are thus put upon the stretch and one has plenty of room for the work. Let your skin incision be a long one.

Leave a fillet of silk in each edge of the tracheal wound to be used in case the inner tube becomes dislodged. Should this accident occur, or should suffocative symptoms arise when the tube is purposely removed, a slight traction upon the silk fillets will open the trachea widely, at once relieving the patient and rendering the reinsertion of the tube easy.

When a lymph gland abscess which has been incised shows indolence in healing the cavity should be frequently curetted and disinfected. When the signs of neighboring gland involvement appear a free and extensive dissection should be undertaken for the removal of all suspicious tissue, whether cicatricial or glandular. A delay of a few days may be permitted where syphilitic disease is suspected in order to try the effect of appropriate general medication.

Catgut may be rendered aseptic and even antiseptic without complicated apparatus by the following simple method: Wind the gut on reels. Shake the full reels in a jar of ether to remove the fat. Soak them in ten per cent. carbolic made with alcohol for from six to twenty-four hours according to the thickness of the gut. Now place the reels in pure alcohol and the catgut is ready for use, and will be found to have lost little or none of its tensile strength.

In using retractors during a dissection be careful to raise up the tissues, not merely separating the lips of the wound, but lifting the skin or fascia away from the deeper structures. In this way the bloodvessels are not emptied and rendered invisible, but may be clearly seen, allowing the operator to secure them before dividing them. The different fascial and muscular layers are also separated from each other, so that they may be divided one at a time. By the faulty method of mere lateral traction the layers rest one upon the other, forming apparently a single membrane, while very considerable vessels, being emptied of their blood, become invisibleand may give one a most disagreeable surprise when, after a stroke of the knife through an apparently innocent bit of fascia, the tension is released and a sudden gush of blood fills the wound.—*International Journal of Surgery.*

